# Effects of culling intensity on diel and seasonal activity patterns of sika deer (*Cervus nippon*)

**DOI:** 10.1038/s41598-019-53727-9

**Published:** 2019-11-20

**Authors:** Takashi Ikeda, Hiroshi Takahashi, Hiromasa Igota, Yukiko Matsuura, Munemitsu Azumaya, Tsuyoshi Yoshida, Koichi Kaji

**Affiliations:** 1 Laboratory of Wildlife Management, United Graduate School of Agricultural Science, Tokyo University of Agriculture and Technology, 3-5-8 Saiwai-cho, Fuchu, Tokyo 183-8509 Japan; 2 Kansai Research Center, Forestry and Forest Products Research Institute, 68 Nagaikyutaroh, Momoyama, Fushimi-ku, Kyoto, Kyoto, 612-0855 Japan; 3 Game Management Laboratory, Department of Environmental and Symbiotic Science, Rakuno Gakuen University, 582 Bunkyodai-Midorimachi, Ebetsu, Hokkaido 069-8501 Japan; 4 Hokkaido Research Center, Forestry and Forest Products Research Institute, 7 Hitsujigaoka, Toyohira-ku, Sapporo, Hokkaido 062-8516 Japan; 5 Yezo Deer Association, South 3 West 21, Chuo-ku, Sapporo, Hokkaido 064-0803 Japan; 6 Wildlife Management Laboratory, Department of Environmental and Symbiotic Science, Rakuno Gakuen University, 582 Bunkyodai-Midorimachi, Ebetsu, Hokkaido 069-8501 Japan; 7 Present Address: Research Center for Wildlife Management, Gifu University, 1-1 Yanagido, Gifu, Gifu, 501-1193 Japan; 8 Present Address: Tohoku Research Center, Forestry and Forest Products Research Institute, 92-25 Nabeyashiki, Shimokuriyagawa, Morioka, Iwate 020-0123 Japan

**Keywords:** Behavioural ecology, Animal behaviour

## Abstract

Adaptive hunting management is commonly used for controlling the populations of overabundant large herbivores; however, induced behavioural changes can make the effective control of target populations difficult. However, few studies have compared the impact of different levels of hunting intensities on the activity patterns of ungulates before, during, and after a culling program. We investigated how different culling intensities affect the activity patterns of sika deer on Nakanoshima Island in Lake Toya, Hokkaido, Japan using camera-trap surveys comparing the period of treatment before, during, and after a culling program. We used the number of deer photographed per hour per camera as an index of activity. Sika deer showed consistent crepuscular activity patterns (i.e., dawn and dusk) during spring–summer and trimodal activity patterns (i.e., dawn, dusk, and midnight) in autumn throughout the study period. In response to increased culling intensity, the activity peaks shifted slightly towards the night. The shift towards nocturnal activity persisted during post-culling period. Understanding the changes in activity patterns in response to culling intensity could be used to facilitate population control and assist in establishing a night shooting program. Thus, wildlife managers should consider night shooting once hunting during day time has shifted the normal diurnal activity of deer to nocturnal activity.

## Introduction

Ungulate species have expanded their range and increased their population size in many areas (Japan^1^; North America^2^; Europe^3^). This has led to an increased number of vehicle collisions^4^, damage to plant communities^5–8^ and agricultural crops^9^. Intensive hunting is commonly used for controlling the populations of overabundant large herbivores, and it is important for adaptive management programs to maintain optimal wildlife populations in order to mitigate potential damage^10^. To achieve this, wildlife managers are required to obtain information on the influence of hunting activity on wildlife behaviour, and use this data for making science-based decisions.

However, induced behavioural changes can make the effective control of target populations difficult. For example, white-tailed deer (*Odocoileus virginianus*) use environments with dense vegetation to avoid hunting predation, making it more difficult to manage the population effectively^11^. Previous studies have also reported that controlled hunting and recreational hunting decreased the harvest availability and observability of white-tailed deer, and suggested that it is important to manage refuge areas and human predation risk on game species in order to increase harvest efficacy and achieve the desired management objectives^12,13^. Among the various impacts of hunting activity on ungulate behaviour, we focused on the activity patterns because ruminants have clear diel activity patterns, which are closely related to their foraging rhythm^14^.

In general, studies have shown that without hunting activity, the activity of white-tailed deer peaked at dawn, dusk, and at night^15^, and the activity of red deer (*Cervus elaphus*) peaked during the day and at night from summer to autumn^16^. On the other hand, it has been indicated that the activity patterns of wildlife generally shift towards nocturnal activity when subjected to human disturbance^17^. Some studies have found an association between hunting activity and the diel activity patterns of ungulates (white-tailed deer^18^, red brocket deer (*Mazama Americana*)^19^, sika deer (*Cervus nippon*) and wild boar (*Sus scrofa*)^20,21^), resulting in increases nocturnal activity during the hunting season and in areas with low protection.

The results of these studies suggest a general change in the activity patterns of large herbivores affected by hunting activity, and we considered that their activity patterns would be greatly and immediately altered by the impact of hunting pressure. However, few studies have compared the impact of different levels of hunting intensities on the activity patterns of ungulates, because it is difficult to control hunting activity and alter hunting programs easily. Although a previous study in Japan reported that crepuscular activity increased with hunting pressure^22^, it is difficult to obtain detailed information on the number of hunted deer, hunting times, hunting sites, and hunting methods. Thus, we conducted a culling program with several levels of culling intensity. Understanding the impact of culling intensity on the activity patterns of deer and their persistence can assist in the establishment of adaptive management programs for wildlife managers. We investigated how different culling intensities affect the activity patterns of sika deer before, during, and after a culling program using camera-trap surveys and hypothesized that the activity patterns of sika deer would change from diurnal to nocturnal activity in response to increased culling intensity.

## Results

The number of deer photographed, camera trap days, and study duration of camera installation on Nakanoshima Island are summarized in Table 1. The total number of deer photographed per trap day gradually decreased as culling progressed from the start of 2012 (Table 1).

**Table 1:** Number of deer photographed in three time periods (day = from 1 h after sunrise to 1 h before sunset, night = from 1 h after sunset to 1 h before sunrise, and twilight = 1 h before and after sunrise and sunset), camera trap days, and study period of camera installation in each year and season (spring = April–May, summer = June–August, and autumn = September–November) on Nakanoshima Island, Hokkaido, Japan, from May to November during 2010 and 2014. Data from 2010 to 2011 are from Ikeda *et al*.^26^.

Year	Season	Day	Night	Twilight	Total	Camera trap days	Study period of camera installation
2010	Spring	—	—	—	—	—	—
	Summer	84	55	72	211	288	August 8 and August 31
	Autumn	413	475	348	1,236	1,074	September 1 and November 30
2011	Spring	269	54	85	408	451	April 22 and May 31
	Summer	731	234	363	1,328	1,033	June 1 and August 31
	Autumn	516	443	322	1,281	997	September 1 and November 30
2012	Spring	428	95	155	678	800	April 22 and May 31
	Summer	871	392	489	1,752	1,778	June 1 and August 31
	Autumn	703	877	559	2,139	1,727	September 1 and November 29
2013	Spring	402	70	211	683	920	April 16 and May 31
	Summer	456	323	351	1,130	1,802	June 1 and August 31
	Autumn	220	478	245	943	1,731	September 1 and November 30
2014	Spring	81	49	117	247	720	April 26 and May 31
	Summer	160	207	190	557	1,812	June 1 and August 31
	Autumn	127	439	198	764	1,684	September 1 and November 30

**Figure 1:**
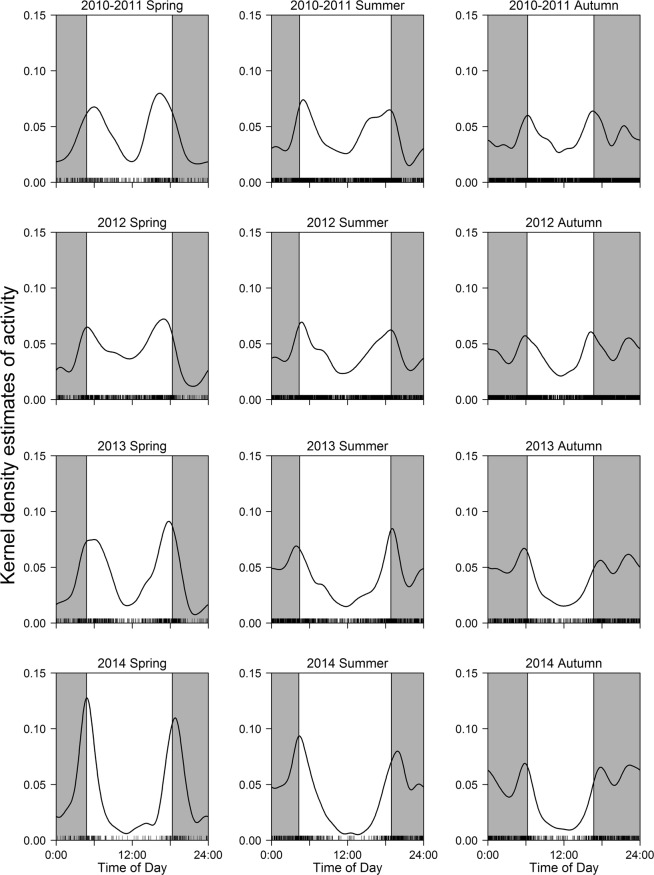
Kernel density estimates of the diel activity patterns of sika deer (Cervus nippon) between summer 2010 and autumn 2014 on Nakanoshima Island, Hokkaido, Japan. Black curves and bars, grey shaded areas, and white areas show kernel density estimates, photo events, night (from seasonal medians of sunset to seasonal medians of sunrise), and day (from seasonal medians of sunrise to seasonal medians of sunset), respectively.

Kernel density estimates of sika deer activity showed crepuscular activity patterns (with peaks around dawn and dusk) during spring and summer, and trimodal patterns (with peaks around dawn, dusk, and midnight) in autumn throughout the study period (Fig. 1). During pre-culling (summer 2010–spring 2012) and low-intensity culling periods (summer 2012–spring 2013), the deer were most active just after sunrise and before sunset in spring and summer and at sunrise and sunset in autumn (Fig. 1). Peak activity, however, shifted towards the night during high-intensity culling (summer 2013–autumn 2013), and this pattern persisted during the post-culling period (spring 2014–autumn 2014) (Fig. 1).

**Figure 2:**
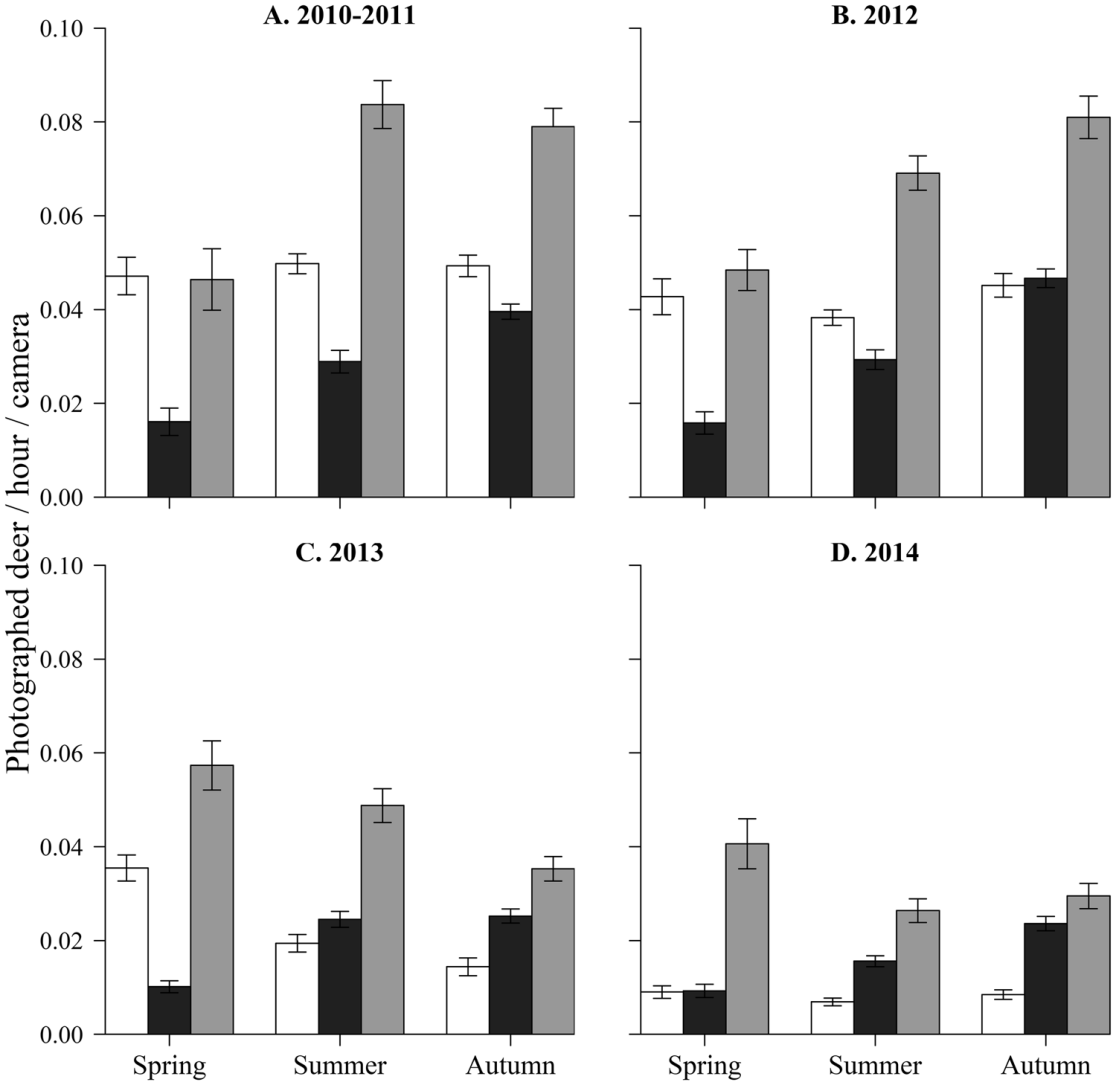
Average daily photographic frequency (the number of deer photographed per hour per camera) and SE for day (from 1 h after sunrise to 1 h before sunset; white), night (from 1 h after sunset to 1 h before sunrise; black), and twilight (1 h before and after sunrise and sunset; grey) in 2010–2011 (**A**), 2012 (**B**), 2013 (**C**), and 2014 (**D**) on Nakanoshima Island, Hokkaido, Japan.

The average daily photographic frequencies during the day, night, and twilight showed distinct changes in deer activity in response to different culling intensity (Fig. 2). The number of deer photographed during twilight was significantly higher than at night throughout the study period (Table 2; Fig. 2A–D). The number of deer photographed during the day was significantly higher than the number photographed at night during the pre-culling and low-intensity culling periods (excluding autumn 2012 and spring 2013), however, it was significantly lower during the high-intensity culling and post-culling periods (excluding spring 2014) (Table 2; Fig. 2A–D). We found no significant differences in the number of deer photographed between night and day in autumn 2012 and spring 2014 (Table 2).

**Table 2:** Estimates (±*SE*) of each explanatory variable for the relationships between total number of deer photographed and three time periods (day = from 1 h after sunrise to 1 h before sunset, night = from 1 h after sunset to 1 h before sunrise, and twilight = 1 h before and after sunrise and sunset) using a generalised linear mixed model with a Poisson distribution.

Year	Season	Intercept		Twilight		Day	
		*Estimates* ± *SE*	*P*	*Estimates* ± *SE*	*P*	*Estimates* ± *SE*	*P*
2010–2011	spring	−4.32 ± 0.20	***	1.10 ± 0.17	***	1.12 ± 0.15	***
	summer	−3.75 ± 0.18	***	1.04 ± 0.08	***	0.53 ± 0.07	***
	autumn	−3.47 ± 0.15	***	0.75 ± 0.05	***	0.27 ± 0.05	***
2012	spring	−4.26 ± 0.15	***	1.12 ± 0.13	***	1.00 ± 0.11	***
	summer	−3.73 ± 0.15	***	0.82 ± 0.07	***	0.25 ± 0.06	***
	autumn	−3.24 ± 0.13	***	0.54 ± 0.05	***	−0.05 ± 0.05	0.32
2013	spring	−4.76 ± 0.17	***	1.75 ± 0.14	***	1.27 ± 0.13	***
	summer	−3.86 ± 0.13	***	0.69 ± 0.08	***	−0.21 ± 0.07	**
	autumn	−4.07 ± 0.19	***	0.35 ± 0.08	***	−0.57 ± 0.08	***
2014	spring	−4.72 ± 0.20	***	1.82 ± 0.15	***	0.12 ± 0.16	0.46
	summer	−4.21 ± 0.15	***	0.59 ± 0.09	***	−0.64 ± 0.09	***
	autumn	−3.99 ± 0.18	***	0.20 ± 0.08	*	−1.06 ± 0.10	***

Parameter estimates of the night period were not shown in this table, because we set the night period as the counterpart of the other time periods (day and twilight). Data are from 12 camera traps during 2010 and 2011 (Ikeda *et al*.^26^) and 20 camera traps during 2012 and 2014 on Nakanoshima Island, Hokkaido, Japan. *P* values indicated significance of differences in the number of deer photographed per hour per camera between night and other time periods, and ***, **, and * showed *P* < 0.001, *P* < 0.01, and *P* < 0.05, respectively.

## Discussion

In this study, we clarified three major characteristics of the activity patterns for sika deer before, during, and after a culling program. First, sika deer showed crepuscular activity patterns with peaks around dawn and dusk during spring and summer, and trimodal activity patterns with peaks around dawn, dusk, and midnight in autumn, throughout the study period. Second, in response to increased culling intensity, the activity peaks shifted slightly towards the night, as nocturnal activity increased and diurnal activity decreased. Third, the shift towards nocturnal activity persisted during the post-culling period.

Sika deer showed trimodal activity patterns in autumn, however, few studies have reported trimodal patterns of ungulate activity. Previous studies have found that the trimodal activity patterns of several ungulates (e.g. elk^23^; alpine chamois (*Rupicapra rupicapra*)^24^; white-lipped deer (*Cervus albirostris*)^25^) are influenced by feeding behaviour, rutting behaviour, and weather conditions. In this study area, however, there was no seasonal consistency between the activity pattern and weather conditions (temperature, precipitation, and wind speed)^26^. Thus, factors such as habitat structure and rutting behaviour may be influencing the trimodal activity patterns observed in sika deer on Nakanoshima Island. As the activity patterns of ungulates are influenced not only by culling intensity, but also feeding behaviour, rutting behaviour, and weather conditions, wildlife managers would have to pay close attention to determine the factor that is causing the change in ungulate activity patterns.

Previous studies in Japan have reported that in protected areas sika deer mainly show a crepuscular activity pattern^27–29^, and a similar activity pattern has also been observed in other deer species (red deer^30,31^; white-tailed deer^15^; moose (*Alces alces gigas*)^32^; roe deer (*Capreolus capreolus*)^33^). In this study area, we found a consistency in the activity patterns with two peaks around dawn and dusk during pre-culling and low-intensity culling periods. From the start of high-intensity culling, on the other hand, activity peaks shifted from dawn and dusk to the night time, and the shift towards nocturnal activity persisted for more than one year after the end of culling.

Another study in Japan reported that the nocturnal activity of sika deer was increased by the presence of agricultural land and forestry area but not by hunting activity^22^. In contrast, a study revealed that sika deer showed nocturnal activity during the non-hunting and hunting seasons, their activity was reduced by hunting activity, and their nocturnal activity increased close to human settlements^21^. In this study, human disturbance affecting deer behaviour was considered to arise only from recreational use and culling programs. Recreational use influenced deer behaviour prior to the study period, while culling programs were conducted only during the years 2012 and 2013. We found no clear changes from diurnal to nocturnal activity under low-intensity culling (i.e., 2.09 days of culling per month, until spring 2013), while high-intensity culling (i.e., 10 days of culling per month) induced changes in the diel activity patterns of sika deer. Thus, it is a possibly that continuous high-intensity culling quickly changes the diel activity patterns and increases nocturnal activity in sika deer, and that the difference in diel activity patterns seen during the pre- and post-culling periods was influenced by high-intensity culling. These findings confirm our hypothesis that the activity patterns of sika deer change from diurnal to nocturnal activity in response to increased culling intensity.

Night shooting was prohibited in Japan prior to 2014. The Ministry of the Environment, however, revised the “Wildlife Protection and Proper Hunting Act” and renamed it “Wildlife Protection and Management, and Proper Hunting Act” in 2014 to promote intensive culling, which allows night shooting under certification requirements. Understanding the changes in activity patterns in response to culling intensity could be used to facilitate population control and assist in establishing a night shooting program. Our results indicate that sika deer shifted to nocturnal activity from diurnal activity because we conducted culling during the day (from sunrise to sunset). Thus, wildlife managers should consider night shooting once hunting during day time has shifted the normal diurnal activity of deer to nocturnal activity. On the other hand, the influence of night shooting on the activity patterns of deer is still unknown and further studies are required to determine these associations before promoting intensive culling.

For more efficient population management, it would be necessary for wildlife managers to conduct culling programs adaptively according to the diel activity patterns of animals, and to also take into account the nocturnal activity of sika deer in the target areas. For example, culling deer at baiting sites during day time and after dark could be an effective measure for population control (e.g., sharpshooting^34,35^). To conduct culling programs effectively, wildlife managers have to recognize the differences in deer response to shooting and trapping. As this study clarified only the influence of culling intensity on diel activity patterns, a future study is required to evaluate the influence of both shooting and trapping on the diel activity pattern.

## Materials and Methods

### Study area

**Figure 3:**
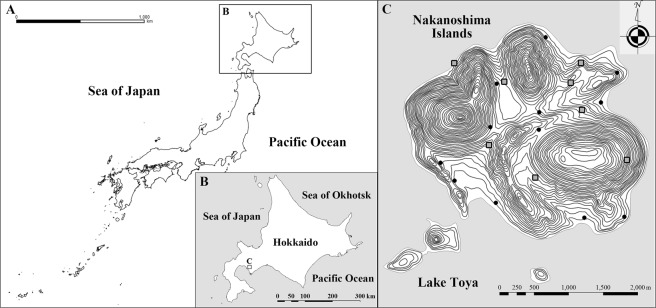
Maps of Nakanoshima Island in Lake Toya, Hokkaido, Japan, showing the location of the twelve camera trap sites (closed circles) between August 2010 and November 2011, and eight new sites (grey squares) that were added to the previous twelve sites between May 2012 and November 2014. Contour lines and the coastline data were obtained from the Geospatial Information Authority of Japan.

We conducted this study on Nakanoshima Island (5.25 km^2^; 42°36′N, 140°51′E, 80–460 m a.s.l.; Fig. 3), Hokkaido, Japan between August 2010 and November 2014. As our study area is located about 4 km offshore, emigration and immigration of deer is negligible. In addition, all parts of this island are designed as a Shikotsu-Tōya National Park, where hunting has been prohibited and there were no predators. As a result of low human disturbance and no hunting or predation, sika deer on this island showed predominantly diurnal activity with two peaks at dawn and dusk between August 2010 and November 2011^34^. The details of our study area have been described in previous studies^26,36^. Population estimates obtained using the drive count method were 44.5 deer/km^2^ (236 deer) and 52.8 deer/km^2^ (277 deer) in March 2011 and March 2012, respectively^37^. The population decreased to 38.7 deer/km^2^ (203 deer) and 14.7 deer/km^2^ (77 deer) in March 2013 and March 2014, respectively, after culling. Vegetation on the island comprises deciduous broad-leaved trees (91.8%), coniferous plantations (6.3%), and open grassland (1.6%). In the deciduous forest, the major canopy species are Japanese oak (*Quercus crispula*), castor-aralia (*Kalopanax pictus*), Japanese bigleaf magnolia (*Magnolia obovata*), painted maple (*Acer mono*), and Japanese linden (*Tilia japonica*)^38^.

### Deer culling

Deer culling was conducted by our study team using various methods such as shotgun, rifle, and several traps. For shotgun and rifle, we culled free ranging deer throughout the study area or attracting deer at bait stations. For trapping, we used corral traps^39^, drop-net traps, and snare traps, and captured the deer at deer trails or by attracting deer using baits. The trapped deer were culled using a shotgun or rifle during the day to ensure the safety of investigators. We adaptively varied the culling sites according to the relative population indexes calculated based on camera trap data, and as a result culled free ranging deer throughout the study area using the above mentioned methods. Additionally, we attracted deer using baits in areas with a low population index. Overall, deer culling was conducted across this study area, regardless of topography and mountain trails. Because deer culling was conducted uniformly throughout the study area during study period, we considered the activity patterns detected by camera traps to correspond to the culling effort.

We conducted culling during the day because night shooting was generally prohibited. Overall, 115 deer were culled during day time, 75 deer during twilight time, and 25 deer during day or twilight time. A total of 53 (corral trap: 22; shotgun and rifle: 28; other methods: 3), 141 (corral trap: 11; shotgun and rifle: 96; snare trap: 26; drop net: 5; other methods: 3), and 21 (corral trap: 3; shotgun and rifle: 17; snare trap: 1) deer were culled in 2012, 2013, and 2014, respectively. Monthly average culling efforts (i.e., the number of culling days) were 2.09 days/month in 2012 and 10 days/month in 2013, respectively, and culling intensity peaked in the summer and autumn of 2013. Similarly, the monthly average number of deer culled was 5.92 deer/month in 2012 and 19.5 deer/month in 2013, respectively, and the cumulative number of deer culled drastically increased starting from summer 2013. Based on culling intensity, we divided the total study period into four culling periods: pre-culling (August 8, 2010–July 31, 2012), low-intensity culling (August 1, 2012–May 31, 2013), high-intensity culling (June 1, 2013–November 30, 2013), and post-culling (April 26, 2014–November 30, 2014).

Deer culling was conducted in accordance with the ethical standards of The Mammal Society of Japan (http://www.mammalogy.jp/en/guideline.pdf). Permission to conduct deer culling in this study was obtained from the Hokkaido Government according to the “Wildlife Protection and Proper Hunting Act” of the Ministry of the Environment, which includes the capture of deer. Permission numbers of the Hokkaido Government permits are: Nos 216–220 from February 1, 2012 to March 31, 2012; Nos 289–293 from April 9, 2012 to March 31, 2013; and Nos 4–21 from April 8, 2013 to March 31, 2014.

### Data collection

We set 20 camera traps (Moultrie Game Spy M-80 Infrared Flash Game Cameras, Cabela’s Inc., U.S.A.) throughout Nakanoshima Island (Fig. 3), including eight new sites that were added to the twelve sites used in a previous study^26^ from May 2012 to November 2014 (Fig. 3). Camera-trap sites were located in the main vegetation type (deciduous broad-leaved trees) of the study area. We set 10 (83.3%) out of 12 cameras during 2010–2011 and 17 (85%) out of 20 cameras during 2012–2014 in this vegetation type. In addition, we set 20 cameras along two fixed routes to determine the herd composition counts of sika deer, which were used successfully to monitor the sex and age ratios^36,40^. Cameras were programmed with a 5 min delay between consecutive groups of photos and three photos were captured per event. Details of our survey design have been published in a previous study^26^. All photos recorded the date and time, and we classified into three time periods: day (from 1 h after sunrise to 1 h before sunset), night (from 1 h after sunset to 1 h before sunrise), and twilight (1 h before and after sunrise and sunset), according to a previous study^41^. We determined the three time periods based on the sunrise and sunset times in the study area^42^. This classification allowed us to examine the effects of culling intensity on the seasonal diel activity patterns of deer. We did not set cameras from December to April because sika deer congregate in wintering areas during this period.

### Data analysis

To quantify the seasonal diel activity patterns of sika deer during 2010–2014 based on the recording times of the photos taken by camera traps, we used kernel density analysis, which is ideal for circular data^43,44^. This method was developed for evaluating the probability density function of a random variable for analysing the activity patterns of carnivores^45^. We defined the seasons as spring (April–May), summer (June–August), and autumn (September–November) based on the vegetation phenology on the island^46^. Because the 2010 survey was limited to August–November, we pooled the data from 2010 and 2011 for the pre-culling period.

To analyse seasonal variations in the diel activity patterns of sika deer, we calculated the average photographic frequency (the number of deer photographed per hour per camera) for all time periods (day, night, and twilight). We tested the differences of the frequencies among the three time periods within each season using a generalised linear mixed model with a Poisson distribution in the glmmML package in R^47^. Because previous studies had suggested that the activity patterns of wildlife tend to shift towards nocturnal activity when subjected to human disturbance^17,21^, we focused on the night period. We defined the three time periods as categorical explanatory variables and set the night period as the counterpart of the other time periods (day and twilight). Thus, the positive beta values estimated by the model indicate that the day and twilight periods had a positive influence on increasing the number of photographs in comparison to the night period. On the other hand, the negative values indicate that these periods had a negative influence compared to the night period. We set the total number of deer photographed for each day, camera, and time period as the response variable and the three time periods as the explanatory variables. Furthermore, we set each camera site as a random factor and the length of each time period (h) as an offset term. Because population size decreased in association with culling from March 2012 to March 2014, we tested the differences in the photographic frequency within each season but not among years and seasons. All statistical analyses were performed using R version 3.1.1^48^.
